# Monitoring seasonal influenza epidemics by using internet search data with an ensemble penalized regression model

**DOI:** 10.1038/srep46469

**Published:** 2017-04-19

**Authors:** Pi Guo, Jianjun Zhang, Li Wang, Shaoyi Yang, Ganfeng Luo, Changyu Deng, Ye Wen, Qingying Zhang

**Affiliations:** 1 Department of Preventive Medicine, Shantou University Medical College, No. 22 Xinling Road, Shantou, Guangdong, 515041, People’s Republic of China

## Abstract

Seasonal influenza epidemics cause serious public health problems in China. Search queries-based surveillance was recently proposed to complement traditional monitoring approaches of influenza epidemics. However, developing robust techniques of search query selection and enhancing predictability for influenza epidemics remains a challenge. This study aimed to develop a novel ensemble framework to improve penalized regression models for detecting influenza epidemics by using Baidu search engine query data from China. The ensemble framework applied a combination of bootstrap aggregating (bagging) and rank aggregation method to optimize penalized regression models. Different algorithms including lasso, ridge, elastic net and the algorithms in the proposed ensemble framework were compared by using Baidu search engine queries. Most of the selected search terms captured the peaks and troughs of the time series curves of influenza cases. The predictability of the conventional penalized regression models were improved by the proposed ensemble framework. The elastic net regression model outperformed the compared models, with the minimum prediction errors. We established a Baidu search engine queries-based surveillance model for monitoring influenza epidemics, and the proposed model provides a useful tool to support the public health response to influenza and other infectious diseases.

Seasonal influenza is a serious public health problem that causes severe illness and death in the world. According to the World Health Organization (WHO), seasonal influenza occurs with an annual attack rate estimated at 5% to 10% in adults and 20% to 30% in children. The epidemics are estimated to result in about 3 to 5 million cases of severe illness and 250,000 to 500,000 deaths worldwide each year^1^. During 2008–2011, an annual average of 92,677 seasonal influenza cases was reported in China^2^. Overall, the influenza pandemics posed a significant burden of excess influenza-associated mortality in the country^3^. To achieve near real-time surveillance of the spread of infectious diseases, several novel approaches based on online surveillance systems and using informal sources such as news reports^4^, social media data^5^,^6^, and search query data^7^,^8^ have been proposed.

In 2009, Ginsberg, J. *et al*.^8^ first presented a novel method of analyzing large numbers of Google search queries to track influenza-like illness in the United States. The proposed method provided near real-time estimates of seasonal influenza activity each day and overcame the limitation of traditional systems requiring 1–2 weeks to gather and process surveillance data^8^. To estimate the seasonal influenza activity and quickly detect outbreaks in China, several programs were used to predict trends of influenza epidemics^9^,^10^. However, these techniques used only influenza-like illness or influenza case data. The robust prediction of influenza epidemics could be improved. In 2013, Yuan, Q. *et al*.^11^ first explored the use of the combination of influenza case data and internet search query data from the search engine Baidu within a linear regression framework to monitor influenza epidemics in China. This provided a new idea to monitor the spread of influenza in the country. To inform the search behavior of users, Baidu released the search volume daily on the Baidu Index website (http://index.baidu.com). The search volume of different search keywords used can be abstracted to assess changes in the search behavior of users.

According to Yuan, Q. *et al*.^11^, the construction of the prediction model involved compositing many search keywords into a single index according to different weights. However, in practice, many search keywords are used to construct the prediction model. The direct compositing of all keywords into a single index is not convenient for assessing the contribution of each keyword to the prediction. Developing robust techniques of search keyword selection and enhancing the ability to predict influenza epidemics remains challenging. Beyond the use of a linear regression model for prediction, we explored an ensemble framework that incorporated different penalized regression algorithms including lasso, ridge and elastic net^12^ to avoid the over-fitting problem with various keywords, identify informative predictors from a pool of candidate keywords, and estimate the parameters of the model with low variability.

In our previous study^13^, use of a penalized regression model based on random bootstrap samples^14^ was able to detect significant variables with better predictive performance. How well a model predicts is practically quantified by performance measures. For example, performance measures such as accuracy, sensitivity, specificity, area under the receiver operating characteristic curve (AUC)^15^ and kappa index of agreement (KIA)^16^ are often used to evaluate performance for classification problems. However, in many settings, the assessment of performance by a single measure has inherent problems^17^. For example, in disease surveillance applications, to predict periods of high incidence of infectious disease requires large sensitivity and/or specificity rates in addition to prediction accuracy^18^. Different performance measures reflect different characteristics of the constructed prediction model. Therefore, under many circumstances, several performance measures must be considered simultaneously.

To improve prediction robustness, we sought to develop a Baidu search engine query data-based prediction model whose performance was optimized with respect to a set of measures. A novel ensemble framework was established by combining bootstrap aggregating (bagging) and a multi-objective optimization method in this study. New ensemble penalized regression models using the lasso, ridge and elastic net algorithms were constructed, and applied to predict seasonal influenza activity. Results of this study indicated that the ensemble elastic net regression model outperformed the compared models in monitoring seasonal influenza activity by using Baidu search engine query data.

## Material and Methods

### Ensemble penalized regression model

#### Penalized regression model

We first considered the lasso (*L1*-penalized regression method) linear regression model^12^. We have an *n* × 1 response vector **y** = (*y*_1_, *y*_2_, …, *y*_*n*_)^*T*^ and linearly independent predictors **x** = (*x*_1*j*_, *x*_2*j*_, …, *x*_*nj*_)^*T*^ (*j* = 1, …, *p*). Let **X** = [**x**_1_, …, **x**_*p*_] be the predictor matrix. We assume that 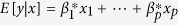. The estimates in the lasso linear regression model are defined as (1):


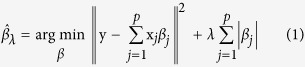


where 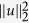 denotes 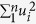 for vector 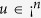, and *λ* is the nonnegative tuning parameter. This estimation method continuously shrinks the coefficients toward 0 as *λ* increases, and some coefficients are shrunk to exactly 0 if *λ* is sufficiently large^19^.

Next, we considered the lasso logistic regression setup by using the tuning parameter *λ*. The estimates 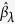 in the model are given by (2):


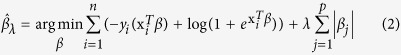


where *λ* is also the tuning parameter used for shrinking coefficients in the model. Generally, the cross-validation method was proposed to select the optimal *λ*^20^. The ridge and elastic net penalized regression models were established using different penalties^12^, and the optimal values of tuning parameters were chose by a similar way.

#### Ensemble penalized regression model built with a bagging strategy

**Figure 1:**
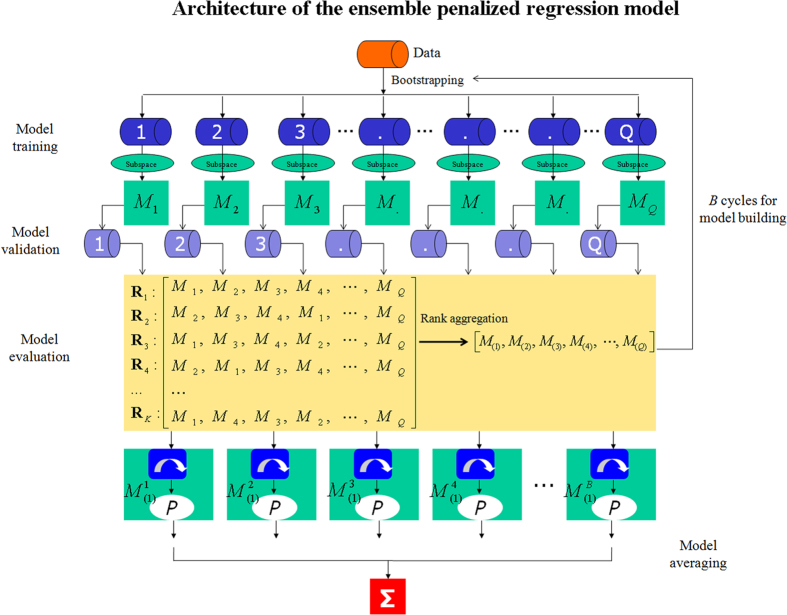
Architecture of the ensemble penalized regression model. A sequence of processing procedures, primarily including model training, validation, evaluation and averaging to be implemented in random bootstrap samplings in this architecture.

To improve the performance of the conventional penalized regression model, we used a combination of bagging and a rank aggregation^21^ method to develop an ensemble penalized regression model. The architecture of the model consists of a sequence of processing procedures primarily including model training, validation, evaluation and averaging, which are implemented in many random bootstrap samplings (Fig. 1). The details for the methodology are presented below.

According to Breiman, L.^22^, bagging is a method of generating multiple versions of a prediction model, and these models are used to obtain an aggregated prediction, which gives substantial gains in prediction accuracy. Suppose that a training set *L* consisting of data *X*_*n*×*p*_ with known outcomes *y* = (*y*_1_, …, *y*_*n*_) that are independently drawn from the probability distribution *P*, then we establish a prediction model *φ*(*X, L*). Here, *n* is the number of samples and *p* is the number of predictors. By taking repeated bootstrap samples {*L*^(*B*)^} from *L*, we formed a set of new prediction models *φ*(*X, L*^(*B*)^). The final prediction of the bagging model denoted by *φ*_*A*_(*x*) = *Eφ*(*X, L*^(*B*)^) was obtained by averaging all results for a number of sub-models. The proof of the validity of bagging on improving prediction accuracy is given in the Methods section of the Supplemental Material.

To build the ensemble model, we randomly drew several (*B*) bootstrap samples from the original data {*X*_*n*×*p*_, *y*_*n*×1_}, trained *B* penalized regression models, *M*^1^, *M*^2^, …, *M*^*B*^, by using the bootstrap samples and combined them to obtain an aggregated prediction. To determine an optimal sub-model in the ensemble penalized regression model according to several performance measures during each random sampling, we used a multi-objective optimization method via the weighted rank aggregation^21^. First, each measure ranked the sub-models according to their performance under that particular measure and generated the ordered lists of sub-models, *R*_*1*_, …, *R*_*K*_, where *K* is the number of measures used. Second, the weighted rank aggregation approach was used to produce an aggregated list that ranked the sub-models according to their performance under all *K* measures simultaneously. To obtain the optimal ordered list of models, we defined the following objective function:


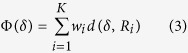


where *δ* is an ordered list of models of size *Q, d* is a distance function that estimates the similarity between any two ordered lists, and *w*_*i*_ is a weight factor associated with each measure. The Spearman footrule distance function^23^ was used to estimate the similarity between any two lists of models.

To determine an optimal model according to all *K* measures simultaneously, it is equivalent to seek out an optimal list δ^*^ to minimize the value of the objective function Φ(*δ*). To determine the optimal parameter δ^*^, the cross-entropy method was used for rank aggregation^24^. The algorithm of the ensemble penalized regression model is given as follows:

**Algorithm.** Ensemble penalized regression model.

**Input:**

- (*X, y*): training set that contains *n* samples and a *p*-dimensional vector of predictors, and 
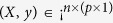
.
- *B*: number of random bootstrap samplings.
- *n*_*bootstrap*_: size of random bootstrap samples with replacement.
- *Q*: size of an ordered list of sub-models in the ensemble model.
- *K*: number of performance measures.
- *RP*: size of random subspace predictor.
- *δ*: an initial ordered list of sub-models of size *L*.
- *d*(.): the Spearman footrule distance function.

**Output:** prediction *ψ*_*average*_ of the ensemble model.

**for** *b* = 1 to *B* **do**

generate bootstrap samples 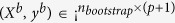


generate out-of-bag (OOB) samples 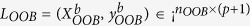


**for** *q* = 1 **to** *Q* **do**

randomly select *RP* predictors as a subset from the original *P* predictors

generate a new subset of predictors 
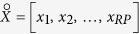


generate new bootstrap samples 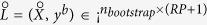


generate new OOB samples 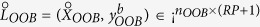


establish a penalized regression model 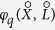


**for** *k* = 1 **to** *K* **do**

compute performance measures *w*_*q*,*k*_ based on OOB samples 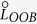


**end**

**end**

generate a matrix of performance measures 
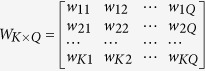
, where the measures in each row (*w*_*i*1_, *w*_*i*2_, …, *w*_*iQ*_) were ranked in order of descending values

generate *K* ordered list of sub-models {*R*_*i*_ = (*M*_1_, *M*_2_, …, *M*_*Q*_)^*i*^, *i* = 1, …, *K*} according to *W*_*K*×*Q*_

establish the objective function 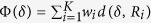
(*w*_*i*_ = (*w*_*i*1_, *w*_*i*2_, …, *w*_*iQ*_))

perform the cross-entropy method for rank aggregation and to determine the optimal parameter δ^*^ minimizing the value of Φ(*δ*)

obtain an optimal ordered list of sub-models 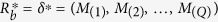


**end**

establish the ensemble penalized regression model according to *B* optimal sub-models 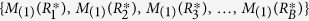


produce the prediction 
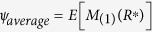
 via model averaging.

From our experience, the model performed similarly when parameter *B* was large, for example, *B* = 100. The value of *n*_*bootstrap*_ was set to the size of the original data. The size of the ordered list of sub-models *Q* was set to 10 to ensure efficiency and fast convergence^21^. Previous studies^25^,^26^ suggested that the random subspace method usually produced an improved ensemble model. Thus, we constructed the ensemble model by using a random subset of predictors, 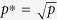, as proposed by Breiman, L.^27^. To assess the contribution of each predictor in the ensemble model, we used a permutation method to estimate the importance of each predictor as follows:


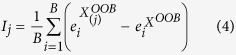


where ***I***_***j***_ is the importance score of predictor *j*, 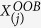 represents the OOB samples with the *j*^*th*^ predictor randomly permuted, ***X***^***OOB***^ is the non-permuted samples, and ***e***_***i***_ is the error rate of prediction. The architecture of the ensemble penalized regression model is depicted in Fig. 1.

### Model evaluation

To widen the application of the ensemble model, we considered two set-ups of the model including the logistic and linear regression models for monitoring influenza epidemics. For the logistic regression model, we used five performance measures, including accuracy, sensitivity, specificity, AUC^15^ and KIA^16^. For the linear regression model, we used relative error (RE), root mean square error (RMSE), mean absolute error (MAE) and symmetric mean absolute percentage error (SMAPE)^28^ to assess performance.

### Application to monitor seasonal influenza activity

#### Data sources

This study used monthly case counts of influenza occurring from January 2011 to May 2015 in China for testing the model. These laboratory-confirmed cases of influenza were reported by physicians to the notifiable disease-monitoring system managed by China’s Center for Disease Control and Prevention, and the data are publicly available on the official website (http://www.moh.gov.cn/). The influenza surveillance data for the studied period corresponded to a total of 53 months of influenza cases. Table 1 shows the details of monthly influenza case counts used in this study.

Search query data were obtained from the Baidu Index website, which contains logs of online search query volume for numerous keywords searched by Baidu users. Since the search query data were available on a daily basis, we converted the data to monthly counts over the study period for analysis.

#### Keyword selection, crawling and filtering

**Figure 2:**
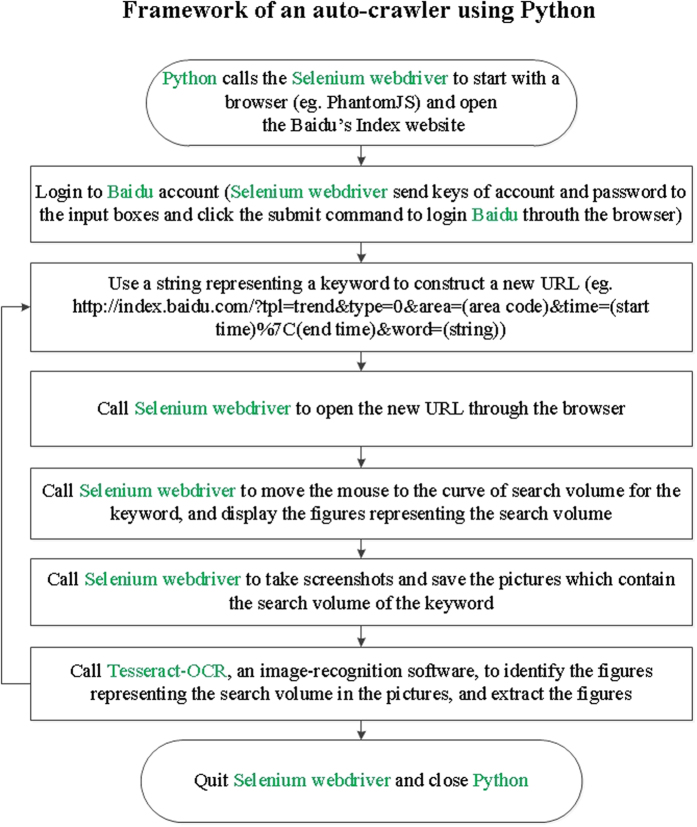
Framework of an auto-crawler using Python to collect search query data from the Baidu Index website. The Selenium webdriver was mainly used with Python for automatic crawling of search query data. The software and search engine used for this analysis are in green.

Previous studies generally chose the names or clinical symptoms of the studied diseases as the primary terms to find more related keywords^11^,^29^,^30^. From this idea, we used the term “influenza” (“
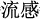
” in Chinese) as a primary keyword to search for more keywords associated with the studied disease on a Chinese website (http://tool.chinaz.com/baidu/words.aspx). The recommended keywords were comprehensively extracted from different sources, including Baidu, portal websites, and blogs^11^. On typing in the primary keyword, a total of 100 related keywords were obtained for further analysis (Table 2). After determining the related keywords, we established an auto-crawler by using Python and used it to collect search volume data for the keywords. The framework of an auto-crawler is depicted in Fig. 2. The Python scripts could be available from the authors for academic usage.

Because some recommended keywords were not necessarily related to influenza epidemics, we further filtered the keywords in three steps: first, the selected search keywords should represent factors that might affect the influenza epidemic; second, the search volume data for each keyword could be presented as a sequential time series with a specific resolution of time (e.g., daily, weekly or monthly); third, the time series of selected keywords should have a maximum cross-correlation coefficient of at least 0.4 with the influenza case data. These filtering approaches were also proposed in previous studies^11^,^30^.

We considered two scenarios of model validation. First, the influenza case surveillance data were divided into a fitting and validation dataset. Models were fitted by using data from January 2011 to June 2014, and the remaining part of the data was used for model validation. Second, to compare the models for monitoring a high level of influenza epidemics, we investigated three cases of high incidence thresholds defined as the median, 75th and 90th percentiles of number of influenza cases over the study period, and evaluated their performance. The receiver operating characteristic (ROC) curve was used to assess the predictive ability of the models.

## Results

On the basis of our filtering steps, 19 of the 100 keywords were not related to influenza epidemics, 8 keywords did not have sequential time series due to low search volume, and a set of only 58 keywords was retained for building the compared models (Table 2). Taking into account the delayed effects of predictors, we considered time lags of 0 to 1 month and the autoregressive term of influenza case number in the previous month. In total, 117 predictors were used for building the prediction models. In this case, the number of predictors was more than the length of time series of influenza cases (117 > 53). Thus, the penalized estimation of parameters in the model was necessary in this study.

In general, influenza causes annual epidemics that peak during the spring and winter in China. Most of our selected search keywords captured the peaks and troughs of the time series curves of influenza cases, so they were good indicators for monitoring influenza epidemics in the country (Figures S1–S5).

**Figure 3:**
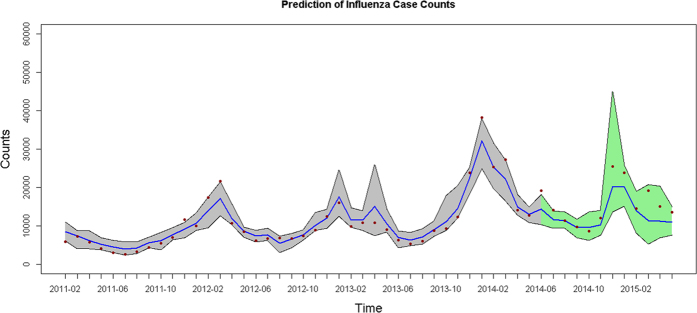
Predictions of influenza cases according to the ensemble elastic net regression model for the period of July 2014 to May 2015. Dark-red dots represent the actual counts of influenza case, blue line represents the fitted counts, and the 95% prediction interval is presented, respectively; the grey areas correspond to the periods used for model fitting and the green areas for prediction.

Comparison of prediction performance of different penalized regression models and the algorithms in the proposed ensemble framework is shown in Table 3. For the prediction of seasonal influenza case counts in the period between July 2014 and May 2015, the ensemble framework improved the performance of the conventional lasso, ridge and elastic net regression models. Among the models, the ensemble elastic net regression model outperformed the others since it had the smallest prediction errors (Table 3). Regardless of the periods for model fitting and prediction, the ensemble elastic net regression model was able to capture the peaks and troughs of the time series curves of influenza cases (Fig. 3). The forecast intervals given by the ensemble model well covered the actual epidemic curve of influenza cases.

**Figure 4:**
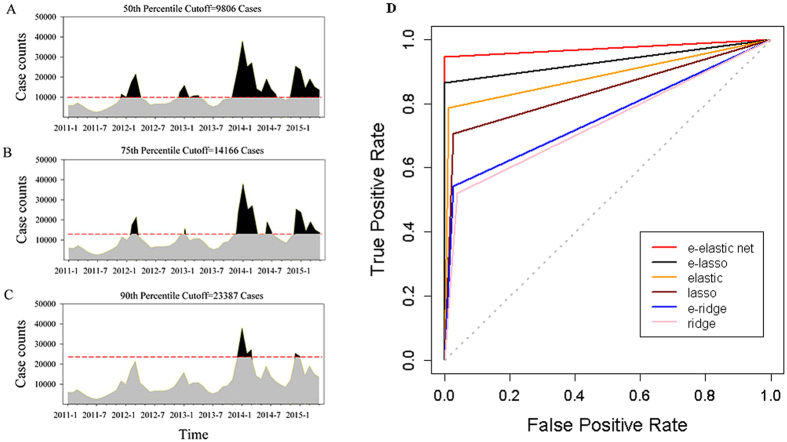
Performance of different penalized regression algorithms (ridge, lasso and elastic net) and the algorithms in the proposed ensemble framework in predicting influenza epidemics. (**A**) High incidence threshold defined as the median percentile of influenza case counts over the study period. (**B**) High incidence threshold defined as the 75th percentile of influenza case counts. (**C**) High incidence threshold defined as the 90th percentile of influenza case counts. (**D**) Comparison of performance of the six prediction models using the receiver operating characteristic (ROC) curve. The e-elastic net, e-lasso and e-ridge models represent the ensemble elastic net, lasso and ridge regression models, respectively.

For monitoring a high level of influenza epidemics, this study integrated the set-up of logistic regression models in the ensemble prediction framework. We studied three situations of high incidence thresholds defined as the median, 75th and 90th percentiles of number of influenza cases over the study period. The performance of the models to detect a large number of influenza cases was assessed using the measures including accuracy, sensitivity, specificity, AUC and KIA (Table 4). Overall, the ensemble elastic net regression model had the largest average AUC of 0.97, and thus outperformed the others, irrespectively of thresholds of influenza incidence used. In addition, it suggested that the predictability of the conventional lasso, ridge and elastic net models was consistently improved by the ensemble framework (Fig. 4).

**Figure 5:**
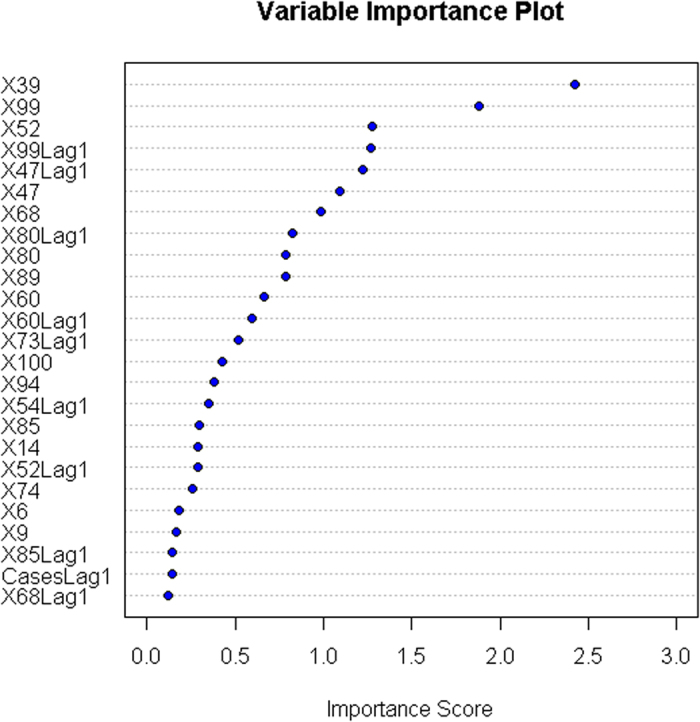
Contribution of each predictor to the prediction in the ensemble elastic net regression model. Only the top 25 significant predictors are shown, and their meanings are described in Table 2. For example, the variable *X39* represents the keyword “*type a flu*” and *X99* denotes the keyword “*saying type a h1n1 flu*”.

Figure 5 shows the estimated importance score for the top 25 keywords contributing to the prediction of the ensemble model. The keyword, “*type a flu*” (variable *X39*), was the most significant factor predicting influenza epidemics over the study period. In addition, the keywords “*saying type a h1n1 flu*” (variable *X99*), “*the toll of swine flu-related death*” (variable *X52*) and “*flu symptom*” (variable *X47*) played important roles in the internet search queries-based surveillance model we established. The ensemble elastic net regression model performed similarly with a large number of random bootstrap samplings, for example, with *B* = 100 (Figure S6). It also guaranteed that the prediction of the ensemble model converged to a stable result.

## Discussion

We used bagging and a multi-objective optimization technology to establish a novel ensemble elastic net penalized regression model to detect seasonal influenza epidemics in China. The results revealed high performance and small fluctuation of extrapolating ability for the proposed model as a Baidu search engine queries-based surveillance framework. The empirical analysis demonstrated that monitoring seasonal influenza epidemics was better with our ensemble models than the conventional penalized regression models.

Recently, Salathé M. *et al*.^31^ discussed the importance of digital disease surveillance for rapid disease outbreak detection and proposed it as a powerful tool to complement traditional approaches. In fact, internet search query data is being explored as a low-cost approach to providing near real-time estimates of disease activity and is becoming widely used for disease surveillance^11^,^18^,^29^,^30^. In China, influenza activity based on routine surveillance data from the ministry of health of China was usually reported with a 1 to 2-week lag. Hence, as a convenient source for timely estimating of influenza activity and detecting an epidemic, search query data can contribute to improve the results of traditional disease surveillance.

In a newly released report^32^, about 87% of Chinese internet users preferred Baidu to search for any information, so it is the most popular search engine in China. With the wide use of the Baidu search engine, the search volume of Baidu naturally reflects Chinese online behavior^30^. Therefore, data from Baidu are more representative of search queries in China for this analysis. Many search keywords are more likely to be captured with this search engine to build a Baidu search engine queries-based surveillance model.

The data for the surveillance model must be automatically fetched over the internet. To achieve this goal, we established an auto-crawler by using Python to collect search volume data for the keywords obtained. The auto-crawler was mainly completed by using the Selenium package within Python. The framework of the auto-crawler included calling the tool of the Selenium webdriver^33^ to start with a browser and open the Baidu Index website, construct a new uniform resource locator (URL) using a keyword, call the Selenium webdriver to open the URL and take screenshots that containing the figures of search volume, and call Tesseract-OCR to extract the data (Fig. 2).

For our empirical analysis, the number of search terms used for predicting influenza epidemics was greater than the sample size (117 > 53) (Table 2). Beyond the use of a linear regression model using a stepwise fashion for significant variable selection and model prediction^11^, this study utilized penalized regression approaches^12^ to establish prediction models with various search keywords. With a large number of predictors in the model, we would prefer to search for a smaller subset that has the strongest effects. A feature of the penalized regression models is a tuning parameter, *λ*, that controls the amount of shrinkage applied to the coefficients. By shrinking variables with very unstable estimates towards zero, the approach can effectively exclude some irrelevant variables and produce a subset of variables with strong effects. Regarding the tuning parameter, the traditional way of choosing the optimal *λ* is to use the cross-validation method. However, the robustness of variable selection is affected by the fold assignment used for cross-validation to some extent^34^. This situation results in estimating the model parameters with a degree of variability. To enhance the predictability of penalized regression models, we combined the methods of bagging and multi-objective optimization to construct the ensemble penalized regression models. Bagging can substantially improve the accuracy of an instable prediction model^22^. Our study suggested that the proposed ensemble framework significantly improved the performance of the conventional lasso, ridge and elastic net regression models, and the ensemble elastic net regression model was optimal in estimating influenza activity.

We found high correlations between specific search terms of Baidu and seasonal influenza incidence. We developed an index of importance score to estimate the contribution of each search term to the prediction of influenza epidemics. Breiman, L.^27^ introduced a practical approach to measure variable importance based on computationally intensive permutations. We adopted this idea and assessed the contribution of each predictor in the ensemble model. For the performance, our predictions of time periods with high influenza incidence based on the ensemble elastic net regression model were very accurate, for different thresholds of high incidence (Table 4). Together, these results demonstrate the viability of the presented ensemble model in supporting influenza surveillance. The ensemble model performed similarly when the number of bootstrap replicates was large. The results of the empirical study indicated that the ensemble model was robust.

Although China has established a notifiable infectious disease monitoring system nationwide, reported influenza cases are available to the public with a delay of about 1 to 2 weeks. The rapid expansion of the geographical distribution and genetic diversity of novel influenza viruses poses a direct challenge to current disease control systems in China^35^. Potentially, influenza may become a long-term threat to public health in this country. Predictive search term-based models were found to perform better than a model using only reported cases to predict future cases^7^,^8^,^11^. Specifically, an internet search-term model returns results more quickly and with better performance^18^. Our study also suggested that most of the selected search keywords captured the peaks and troughs of the time series curves of influenza cases. Our ensemble elastic net regression model predicted seasonal influenza epidemics with high performance. Thus, in China, this internet search term-based system might be used as a supplement to existing surveillance systems. However, we should note that surveillance models based on internet search query data like Google Flu Trends have substantial flaws including missing the first wave of the 2009 influenza H1N1 pandemic and overestimating the intensity of the H3N2 epidemic during the 2012/2013 season in United States^36^. It means that there is room to improve the performance of surveillance models based on internet search query data and provide reliable surveillance for seasonal or pandemic influenza^36^. In addition, because Google has pulled out of mainland China since 2010, search query data from Google during the study time period of 2011–2015 are not publicly available in mainland China. Therefore, an overall comparison between the algorithm proposed in this study and that of Google Flu Trends cannot be made. All of these drive us to further validate the performance of the proposed algorithm by ongoing studies in the future.

Several limitations of this study should be mentioned. In fact, different people may use different words to search for the same information, especially when searching in Chinese, which has various ways of expression. Thus, search keywords should be carefully selected to reflect terms most likely associated with influenza epidemics. As well, internet searching behavior was susceptible to the impact of media reports, which might affect the performance of the internet search term-based system^37^. Third, in the empirical study, 100 bootstrap replicates were used for building the ensemble model. With this setting, the ensemble prediction was converged to a stable result but required much time to generate an aggregated prediction. This issue was also discussed by Breiman, L.^27^. A procedure for parallel computing integrated into the ensemble model to speed up the analysis would be practical. Hence, the computing efficiency needs to be improved.

In conclusion, this present study developed a novel ensemble elastic net penalized regression model by combining bagging and a multi-objective optimization method to monitor seasonal influenza activity. The approach provided a useful tool in support of the public health response to influenza and other infectious diseases in China.

## Additional Information

**How to cite this article:** Guo, P. *et al*. Monitoring seasonal influenza epidemics by using internet search data with an ensemble penalized regression model. *Sci. Rep.* **7**, 46469; doi: 10.1038/srep46469 (2017).

**Publisher's note:** Springer Nature remains neutral with regard to jurisdictional claims in published maps and institutional affiliations.

## Supplementary Material

Supplementary Material

## Figures and Tables

**Table 1:** Data of influenza cases confirmed by laboratory test for the period January 2010 to May 2015 in China were publicly available from China’s Center for Disease Control and Prevention.

Month	Cases	Month	Cases	Month	Cases	Month	Cases	Month	Cases
2011–01	6072	2011–12	11631	2012–11	8942	2013–10	9309	2014–09	9751
2011–02	5930	2012–01	10046	2012–12	12411	2013–11	12317	2014–10	8635
2011–03	7299	2012–02	17421	2013–01	16012	2013–12	23894	2014-11	12043
2011–04	5727	2012–03	21625	2013–02	9806	2014–01	38214	2014–12	25477
2011–05	4130	2012–04	10707	2013–03	10761	2014–02	25279	2015–01	23828
2011–06	3065	2012–05	8520	2013–04	10844	2014–03	27262	2015–02	14480
2011–07	2654	2012–06	6195	2013–05	9006	2014–04	14166	2015–03	19199
2011–08	3243	2012–07	6738	2013–06	6254	2014–05	12685	2015–04	15063
2011–09	4360	2012–08	6793	2013–07	5338	2014–06	19220	2015–05	13625
2011–10	5525	2012–09	6762	2013–08	6098	2014–07	14056		
2011–11	7055	2012–10	7331	2013–09	8751	2014–08	11419		

**Table 2:**
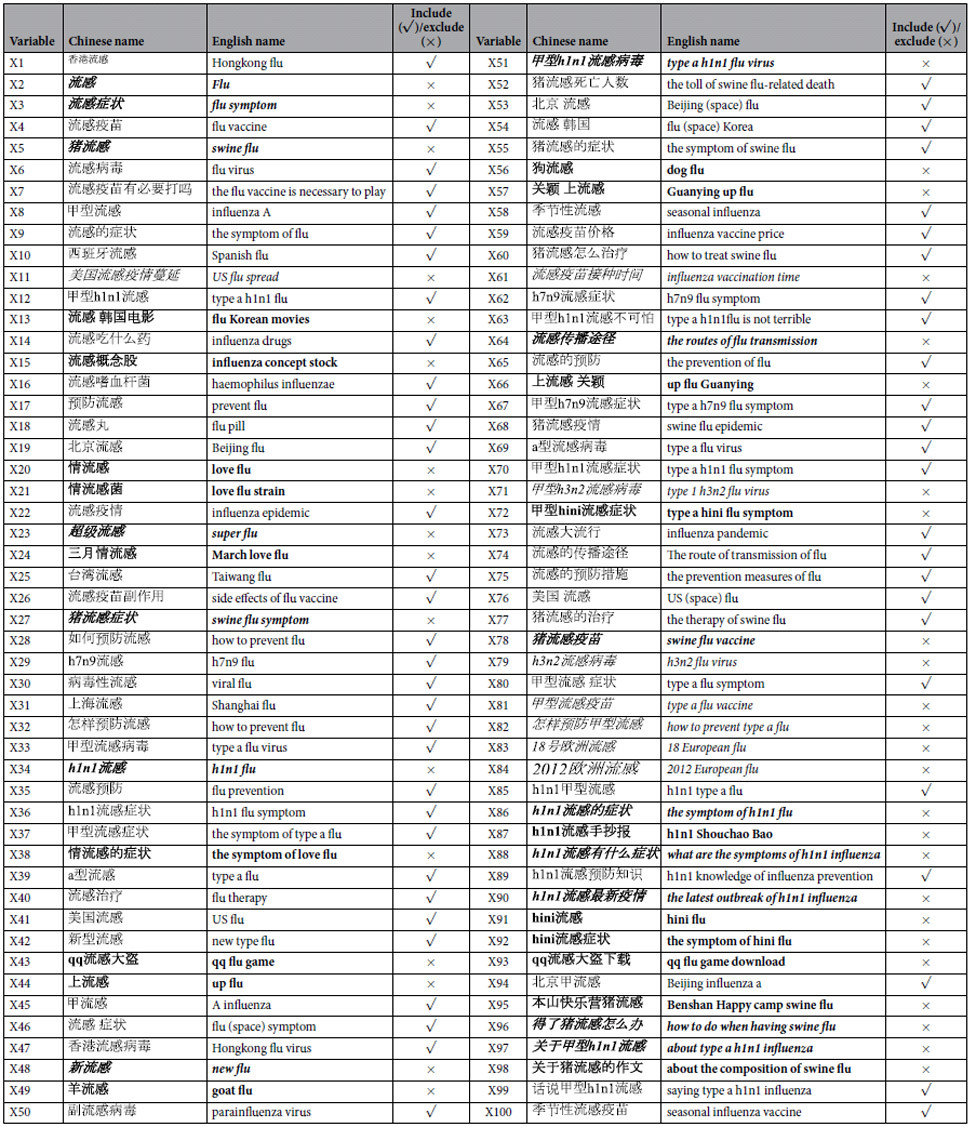
Search keywords from Baidu search engine used in this study.

The search terms in bold were excluded at filtering step (i), search terms in italics were excluded at filtering step (ii) and search terms in bold and italics were excluded at filtering step (iii). Step (i): the selected search terms should represent factors that might influence the influenza epidemics. Step (ii): the search query data for each term was a sequential time series with a daily, weekly or monthly resolution. Step (iii): the time series of selected search terms should have a maximum cross-correlation coefficient of at least 0.4 with the influenza case data.

**Table 3:** Prediction performance of different penalized regression algorithms (lasso, ridge and elastic net) and the algorithms in the proposed ensemble framework was compared using the number of influenza cases during the period of July 2014 to May 2015.

Prediction period	Model	RMSE	MAE	RE	SMAPE
2014/07–2015/05	ridge	5283.87	4045.57	26.41%	26.53%
	lasso	4702.71	3419.20	25.16%	23.60%
	elastic net	3396.04	2799.94	21.29%	21.19%
	ensemble ridge	4148.28	3285.35	22.73%	21.74%
	ensemble lasso	3756.31	2897.91	20.42%	19.24%
	ensemble elastic net	3488.42	2650.17	19.09%	17.55%

Measures including relative error (RE), root mean square error (RMSE), mean absolute error (MAE) and symmetric mean absolute percentage error (SMAPE) were used to assess the predictions.

**Table 4:** Comparison of different penalized regression algorithms (ridge, lasso and elastic net) and the algorithms in the proposed ensemble framework in predicting influenza epidemics, by using three cases of high incidence thresholds defined as the median, 75th and 90th percentiles of number of influenza cases over the study period.

Percentile cutoff	Model	Acc	Sen	Spe	AUC	KIA
median	ridge	0.92	0.96	0.88	0.92	0.85
	lasso	0.92	0.93	0.92	0.92	0.85
	elastic net	0.98	1.00	0.96	0.98	0.96
	ensemble ridge	0.94	0.96	0.92	0.94	0.88
	ensemble lasso	1.00	1.00	1.00	1.00	1.00
	ensemble elastic net	1.00	1.00	1.00	1.00	1.00
75th	ridge	0.85	0.43	1.00	0.71	0.52
	lasso	0.96	0.86	1.00	0.93	0.90
	elastic net	0.96	0.86	1.00	0.93	0.9
	ensemble ridge	0.87	0.50	1.00	0.75	0.59
	ensemble lasso	0.98	0.93	1.00	0.96	0.95
	ensemble elastic net	1.00	1.00	1.00	1.00	1.00
90th	ridge	0.90	0.17	1.00	0.58	0.26
	lasso	0.92	0.33	1.00	0.67	0.47
	elastic net	0.94	0.50	1.00	0.75	0.64
	ensemble ridge	0.90	0.17	1.00	0.58	0.26
	ensemble lasso	0.96	0.67	1.00	0.83	0.78
	ensemble elastic net	0.98	0.83	1.00	0.92	0.90

Measures including accuracy (Acc), sensitivity (Sen), specificity (Spe), area under the receiver operating characteristic curve (AUC) and kappa index of agreement (KIA) were used to assess the predictions.
