# Supplementary material for: Monitoring seasonal influenza epidemics by using internet search data with an ensemble penalized regression model
Source: Sci Rep. 2017 Apr 19;7:46469. doi: 10.1038/srep46469 (PMC5396076; doi:10.1038/srep46469)

**Supplemental Material**

**Monitoring seasonal influenza epidemics by using internet search data with an ensemble penalized regression model**

Pi Guo, Jianjun Zhang, Li Wang, Shaoyi Yang, Ganfeng Luo, Changyu Deng, Ye Wen, Qingying Zhang^*^

Department of Preventive Medicine, Shantou University Medical College, No. 22 Xinling Road, Shantou, Guangdong 515041, People's Republic of China

*** Corresponding author**

Dr. Qingying Zhang

Department of Preventive Medicine, Shantou University Medical College, Shantou, Guangdong 515041, China

Telephone: 086-0754-88566774

Fax number: 86-754-88566774

E-mail: [qyzhang@stu.edu.cn](mailto:qyzhang@stu.edu.cn)

**Appendix Content**

Section Page

| Methods | The proof of the validity of bagging method on improving the prediction. | 3 |
| --- | --- | --- |
| Figure S1 | Time series curves of influenza case and search keywords (*X1*, *X4*, *X6*, *X7*, *X8, X9*, *X10*, *X12*, *X14*, *X16*, *X17*, *X18*) from January 2011 to May 2015. | 5 |
| Figure S2 | Time series curves of influenza case and search keywords (*X19*, *X22*, *X25*, *X26*, *X28, X29*, *X30*, *X31*, *X32*, *X33*, *X35*, *X36*) from January 2011 to May 2015. | 6 |
| Figure S3 | Time series curves of influenza case and search keywords (*X37*, *X39*, *X40*, *X41*, *X42, X45*, *X46*, *X47*, *X50*, *X52*, *X53*, *X54*) from January 2011 to May 2015. | 7 |
| Figure S4 | Time series curves of influenza case and search keywords (*X55*, *X58*, *X59*, *X60*, *X62, X63*, *X65*, *X67*, *X68*, *X69*, *X70*, *X73*) from January 2011 to May 2015. | 8 |
| Figure S5 | Time series curves of influenza case and search keywords (*X74*, *X75*, *X76*, *X77*, *X80, X85*, *X89*, *X94*, *X99*, *X100*) from January 2011 to May 2015. | 9 |
| Figure S6 | Assessment of the effect of cycling parameter *B* (number of random bootstrap samplings) on the prediction of ensemble elastic net penalized regression model. | 10 |

**Methods**

Below we present the effectiveness of the bagging method on improving the final prediction. Suppose *y* is numerical and is the prediction model. The aggregated prediction is . Take *Y*, *X* to be random variables having distribution *P*. The average prediction error *e* in is

Define the error *e_A_* in the aggregated prediction to be

By using the Schwarz inequality $(EZ)^{2}\leq EZ^{2}$, we obtain the following proof:

Thus, has lower mean-squared prediction error than . This result suggests that the method of bagging provided substantial gains in prediction accuracy.

Suppose *y* is categorical and a prediction model predicts a class label $j\in\{1,...,J\}$. Assume that *L* is drawn from the distribution *P*, and *Y*, *X* are from *P*, then the probability of correct prediction for *L* fixed is:

Denote , then the averaged probability of correct prediction is

$$\begin{matrix} r=\sum_{j} E(Q(j|X)|Y=j)P(Y=j) \\ \text{ }=\sum_{j} \int Q(j|x)P(j|x)P_{X}(dx) \end{matrix}$$

where $P_{X}(dx)$ is the overall *x* distribution.

Since ,

$$r_{A}=\sum_{j} \int I(\arg\max_{i} Q(i|x)=j)P(j|x)P_{X}(dx)$$

where $I(\cdot)$ is the indicator function. Consider the set

$$C=\{x;\arg\max_{j} P(j|x)=\arg\max_{j} Q(j|x)\}$$

For $x\in C$

$$\sum_{j} I(\arg\max_{i} Q(i|x)=j)P(j|x)=\max_{j} P(j|x)$$

so that

Prior to the bagging prediction, the highest correct prediction rate is $r^{*}=\int\max_{j} P(j|x)P_{X}(dx)$, which is given by the prediction

$$Q^{*}(x)=\arg\max_{j} P(j|x)$$

Thus, we get $r_{A}\geq r^{*}$. The bagging method substantially increased the probability of correct prediction.

**Figure S1. Time series curves of influenza case and search keywords (*X1*, *X4*, *X6*, *X7*, *X8, X9*, *X10*, *X12*, *X14*, *X16*, *X17*, *X18*) from January 2011 to May 2015.**





**Figure S2. Time series curves of influenza case and search keywords (*X19*, *X22*, *X25*, *X26*, *X28, X29*, *X30*, *X31*, *X32*, *X33*, *X35*, *X36*) from January 2011 to May 2015.**





**Figure S3. Time series curves of influenza case and search keywords (*X37*, *X39*, *X40*, *X41*, *X42, X45*, *X46*, *X47*, *X50*, *X52*, *X53*, *X54*) from January 2011 to May 2015.**





**Figure S4. Time series curves of influenza case and search keywords (*X55*, *X58*, *X59*, *X60*, *X62, X63*, *X65*, *X67*, *X68*, *X69*, *X70*, *X73*) from January 2011 to May 2015.**





**Figure S5. Time series curves of influenza case and search keywords (*X74*, *X75*, *X76*, *X77*, *X80, X85*, *X89*, *X94*, *X99*, *X100*) from January 2011 to May 2015.**





**Figure S6. Assessment of the effect of cycling parameter *B* (number of random bootstrap samplings) on the prediction performance of the ensemble elastic net penalized regression model.** Monthly data of influenza cases corresponding to the period from January 2011 to June 2014 were used for model training, and data corresponding to the period from July 2014 to May 2015 were for model evaluation.


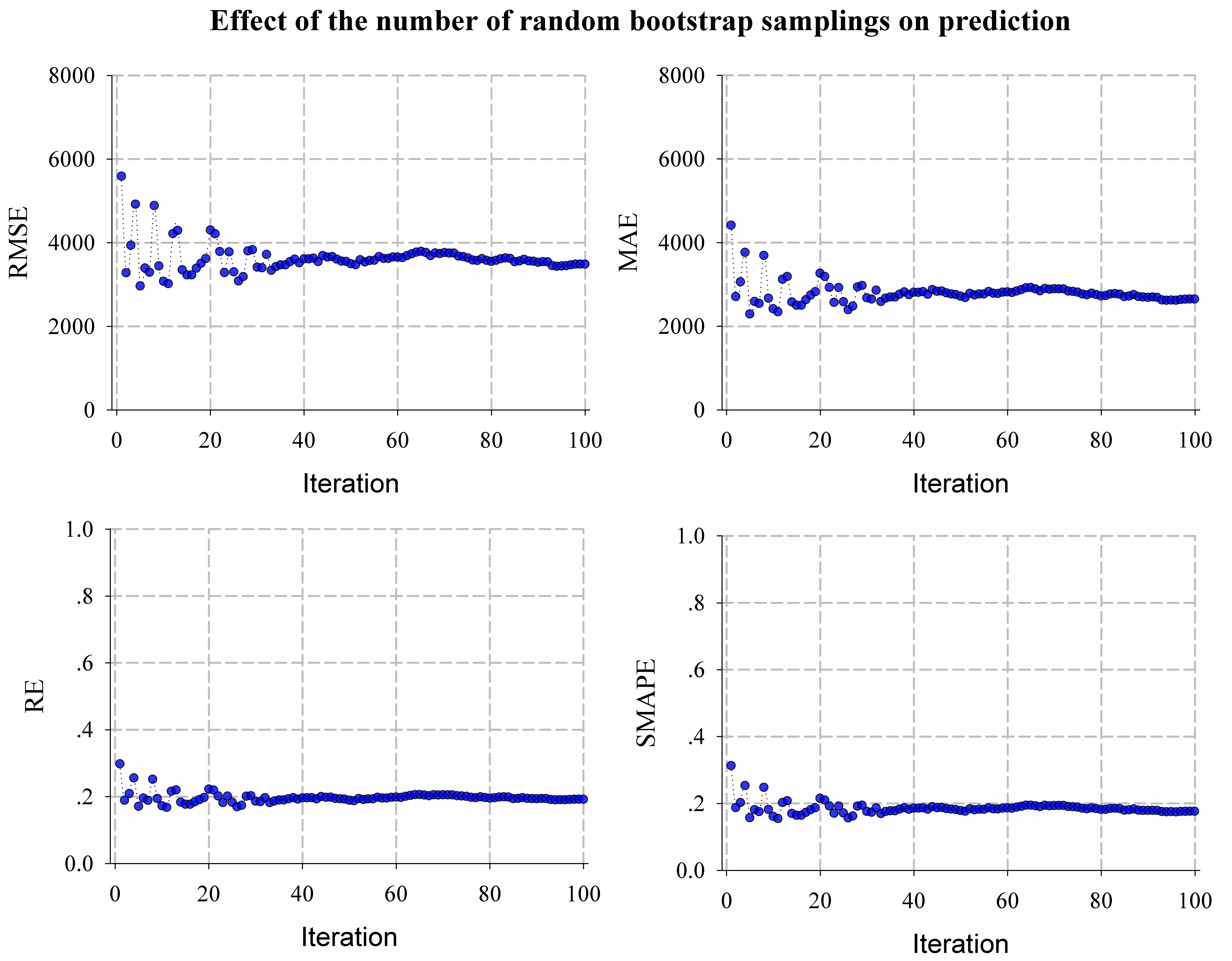

Supplement: Supplementary Material [file srep46469-s1.doc]
